# Mitochondrial Transplantation in Animal Models of Psychiatric Disorders: A Novel Approach to Psychiatric Treatment

**DOI:** 10.3390/biom15020184

**Published:** 2025-01-27

**Authors:** Keiko Iwata, Masafumi Noguchi, Norihito Shintani

**Affiliations:** Laboratory of Pharmacology, School of Pharmaceutical Sciences, Wakayama Medical University, 25-1 Shichibancho, Wakayama 640-8156, Japan

**Keywords:** psychiatric disorders, brain functions, experimental disease models, mitochondrial transplantation, therapeutic strategies

## Abstract

Mitochondria are essential for brain function, and accumulating evidence from postmortem brain studies, neuroimaging, and basic research indicates mitochondrial impairments in patients with psychiatric disorders. Restoring mitochondrial function therefore represents a promising therapeutic strategy for these conditions. Mitochondrial transplantation, an innovative approach that uses functional mitochondria to repair damaged cells, has demonstrated efficacy through various delivery methods in cell, animal, and animal disease models. This review explores the critical link between mitochondria and psychiatric disorders and provides an overview of mitochondrial transplantation as a therapeutic intervention. It highlights recent advances in mitochondrial transplantation in animal models of psychiatric disorders, focusing on delivery methods, the timing of administration, and the integration of exogenous mitochondria into brain cells. The potential therapeutic effects and the mechanisms that underlie these effects are discussed. Additionally, this review evaluates the clinical relevance, challenges, and future strategies for the application of mitochondrial transplantation in the treatment of psychiatric disorders.

## 1. Introduction

Psychiatric disorders, including major depressive disorder (MDD) and schizophrenia, are complex conditions characterized by disturbances in cognition, perception, emotions, behavior, and social functioning. MDD is primarily defined by persistent sadness, anhedonia (loss of interest or pleasure), feelings of guilt or low self-worth, cognitive impairments such as poor concentration, and disruptions in sleep and appetite. Patients often experience fatigue and psychomotor slowing. Schizophrenia, in contrast, encompasses both psychotic and non-psychotic symptoms. Psychotic symptoms include hallucinations (sensory experiences without external stimuli), delusions (firmly held, false beliefs resistant to contrary evidence), and thought disorders (disorganized or abnormal thought patterns). Non-psychotic symptoms include blunted affect (reduced emotional expression), avolition (diminished motivation to pursue goals), impaired social functioning, motor abnormalities, and cognitive deficits affecting memory, attention, and executive functioning.

Treatment response rates emphasize the necessity for improved therapeutic strategies: only about 50% of individuals with MDD experience symptom improvement with antidepressant treatment, and a mere 30% achieve full remission [1]. Similarly, in schizophrenia, while antipsychotics are effective in alleviating acute psychotic and disorganized symptoms [2], treatment remains incomplete, highlighting the critical need for novel approaches in managing psychiatric disorders. The etiology of these disorders involves the complex interplay of genetic predispositions and environmental stressors, such as prenatal factors, trauma, and psychosocial stress, which may disrupt neurodevelopmental trajectories and increase susceptibility to mental illness later in life. Additionally, multiple human studies have identified mitochondrial dysfunction as a key feature in psychiatric disorders [3].

In recent years, mitochondrial transplantation and its potential clinical applications have gained substantial attention [4]. Evidence is accumulating from studies using experimental animal models on the effects of mitochondrial transplantation in central nervous system disorders [5]. This review specifically focuses on mitochondrial transplantation in models of psychiatric disorders, such as MDD and schizophrenia.

## 2. Mitochondrial Transplantation and Its Therapeutic Applications

Mitochondrial transplantation is emerging as a novel therapeutic approach for mitochondrial diseases, which are a group of genetic disorders characterized by dysfunctional mitochondria, the cell’s energy-producing organelles. These diseases can affect multiple organs, particularly those with high energy demands such as the brain, heart, and muscles, leading to severe symptoms including muscle weakness, neurodegeneration, and organ failure. Mitochondrial transplantation has gained attention for its potential to replace defective mitochondria in affected cells, thereby restoring cellular function and alleviating disease symptoms [4].

Mitochondrial transplantation involves isolating healthy, functional mitochondria from donor cells and introducing them into cells with mitochondrial dysfunction. Mitochondria are dynamic organelles capable of fusion and fission, allowing them to form interconnected networks. Through fission, cells can isolate and remove damaged mitochondria, effectively mitigating cellular stress. At the same time, fusion allows for the sharing of functional components to support and complement dysfunctional mitochondria [6]. Mitochondrial transplantation introduces exogenous mitochondria that can fuse with the recipient cells’ endogenous mitochondria. This fusion has been observed to enhance oxygen consumption rates, increase ATP production, and restore depleted mitochondrial DNA [7,8].

Studies in cell cultures and animal models indicate the potential of mitochondrial transplantation as an emerging therapeutic approach. In neurological models, mitochondrial transplantation has shown potential in reducing neuronal damage and improving motor function, suggesting potential applications in neurodegenerative diseases [9,10,11,12,13]. Clinically, this approach has shown promise in treating myocardial ischemia–reperfusion injury and cardiogenic shock. Emani et al. applied autologous mitochondrial transplantation in pediatric patients with myocardial ischemia–reperfusion injury post-surgery, where four out of five patients showed improved cardiac function and were successfully weaned from extracorporeal membrane oxygenation [14]. A retrospective study further supported these findings, with significantly higher extracorporeal membrane oxygenation independence in patients receiving mitochondrial transplantation [15].

## 3. Mitochondria and Their Importance for Brain Functions

Mitochondria are known as the “powerhouses” of cells because of their central role in producing ATP (adenosine triphosphate) through oxidative phosphorylation, which is essential for sustaining cellular functions. The brain has high energy demands; the brain accounts for only 2% of body weight, but consumes approximately 20% of the body’s total energy, primarily to maintain neuronal signaling, synaptic transmission, and ion gradients across cell membranes [16]. Mitochondria supply ATP to synapses to support synaptic vesicle cycling by controlling synaptic Ca^2+^ homeostasis, neurotransmitter release, and receptor functions essential for synaptic plasticity, a process underlying learning and memory. Additionally, the presence of mitochondria at synapses is critical for regulating local energy demands during neuronal activity [17,18]. Mitochondria also produce reactive oxygen species (ROS) as a byproduct of ATP production. While low levels of ROS play a signaling role, excessive ROS can damage lipids, proteins, and DNA within cells. Mitochondria have intrinsic antioxidant systems to neutralize ROS, which protects neurons from oxidative damage. Impaired ROS management in mitochondria is linked to psychiatric disorders [19,20]. In addition, mitochondria influence brain development by regulating the differentiation, proliferation, and maturation of neural cells [21]. Disruptions in mitochondrial function during development can impair the formation of neural circuits and increase the risk of psychiatric disorders, indicating that mitochondrial health is essential from early life stages.

## 4. Mitochondrial Impairments in Patients with Psychiatric Disorders

Neuroimaging studies have underscored mitochondrial abnormalities in psychiatric disorders. Phosphorus-31 magnetic resonance spectroscopy (^31^P-MRS) can assess regional high-energy phosphorus compounds, providing insight into mitochondrial function. In some ^31^P-MRS studies of schizophrenia, reduced ATP levels were observed in the frontal and temporal lobes as well as the basal ganglia [22,23,24]. Studies in both MDD and schizophrenia have also demonstrated decreased levels of N-acetylaspartate, an indicator of neuronal mitochondrial activity that reflects neuronal density and mitochondrial health [25,26].

Glucose metabolism in psychiatric disorders has also been studied through positron emission tomography with ^18^F-fluoro-deoxy-glucose. In MDD, reduced metabolic rates are reported in the prefrontal cortex, anterior cingulate cortex, and caudate nucleus, along with decreased glucose metabolism in regions including the bilateral insula, left putamen, right caudate, and cingulate gyrus [27,28]. In schizophrenia, negative symptoms are associated with hypoactivity and hypometabolism in the frontal, prefrontal, and anterior cingulate cortices, while positive symptoms are linked to hypermetabolism in the temporolimbic system, including the amygdala, basal ganglia, and temporal cortex [29].

Postmortem studies of schizophrenia have further revealed marked mitochondrial abnormalities, especially in oligodendroglial cells within the caudate nucleus and the prefrontal cortex, where decreased mitochondrial number and volume density are observed compared with controls [30]. Additionally, schizophrenia patients display significantly fewer mitochondrial profiles in both the caudate and putamen than healthy controls [31].

In summary, these findings collectively indicate that mitochondrial dysfunction and abnormalities in energy metabolism are prominent features across MDD and schizophrenia.

## 5. Animal Models of Psychiatric Disorders Used for Mitochondrial Transplantation

### 5.1. Animal Models of MDD

Several animal models of MDD have been developed, including the chronic restraint stress model, the chronic mild stress model, the lipopolysaccharide model, the social defeat model, and the learned helplessness model [32]. Mitochondrial transplantation studies have used three different MDD animal models [33,34,35]. A common feature among these models is the involvement of inflammatory cytokines in the development of depressive behaviors. Each of these MDD animal models is described in detail below.

#### 5.1.1. Lipopolysaccharide (LPS) MDD Model

The LPS-induced MDD model is extensively used to explore the relationship between inflammation and depression-like behaviors. LPS, a component of bacterial cell walls, is administered to provoke an immune response, resulting in systemic inflammation. This inflammatory state mimics the behavioral and biochemical features of MDD observed in humans. In this model, depression-like behaviors correlate with the release of pro-inflammatory cytokines, including interleukin 1β (IL1β), interleukin 6 (IL6), and tumor necrosis factor α (TNFα), in both the brain and plasma, thereby establishing a connection between inflammation and depressive symptoms [36,37,38]. Additionally, LPS induces microglial activation in the brain [39]. Western blot analyses of LPS-treated mice have demonstrated marked upregulation of NLRP3 and pro-IL1β expression via the NFκB pathway, accompanied by neuronal degeneration in the hippocampus [38].

Mitochondrial dysfunction is another hallmark of this model. LPS exposure causes neuronal damage, particularly in the hippocampus, with the increased expression of inflammatory markers such as heme oxygenase 1, which exacerbates oxidative stress [36]. Furthermore, LPS exposure induces pyroptosis in the hippocampus and results in mitochondrial dysfunction, characterized by mitochondrial swelling, disrupted cristae structure, reduced mitochondrial membrane potential, diminished ATP production, and elevated ROS levels in hippocampal neurons [34,40].

#### 5.1.2. Chronic Restraint Stress (CRS) MDD Model

Restraint stress is a well-established experimental model for inducing depressive-like behaviors in rodents. For CRS, animals are typically restrained for at least 2 h daily over 14 to 21 days. This stress paradigm induces a variety of phenotypes, including damage or atrophy of hippocampal CA3 pyramidal neurons [41,42,43], hyperactivation of the hypothalamic–pituitary–adrenal (HPA) axis [44], depressive-like behaviors, and apoptotic cell death [45]. In the hippocampus of CRS-model animals, levels of pro-inflammatory cytokines, such as IL1β, IL6, COX2, and TNFα, are significantly elevated, whereas levels of anti-inflammatory factors, such as IL10 and IL4, are markedly reduced [46]. Additionally, CRS can dysregulate GABAergic neurotransmission in the hippocampus by enhancing caspase 1-mediated neuroinflammation, which ultimately contributes to the emergence of depression-like behaviors [47].

CRS also impairs mitochondrial function by inhibiting Na⁺/K⁺-ATPase activity and suppressing the activity of mitochondrial respiratory chain complexes I, III, and IV in the brain regions involved in mood regulation, contributing to the bioenergetic deficits characteristic of depressive states [48]. Additionally, CRS is associated with reduced ATP levels in the prefrontal cortex [33].

#### 5.1.3. Aged Chronic Mild Stress (CMS) MDD Model

The CMS paradigm is widely employed as a model for investigating antidepressant efficacy and the pathophysiology of major depressive disorder (MDD) [49]. In this model, rodents are exposed to unpredictable mild stressors, such as food and water deprivation, cage tilt, wet bedding, white noise, and strobe lighting, for at least four weeks. Sustained exposure to CMS activates the HPA axis, resulting in significant neurotoxic effects on the hippocampus and prefrontal cortex, both of which are crucial in regulating HPA activity. Normally, the hippocampus provides negative feedback to the HPA axis; however, chronic glucocorticoid exposure damages hippocampal neurons, induces neuroinflammation, and activates microglia [50,51]. This damage diminishes hippocampal inhibitory feedback, exacerbating HPA overactivity. Similarly, pyramidal neurons in the prefrontal cortex, another key regulator of the HPA axis, undergo stress-induced dendritic atrophy, leading to reduced prefrontal cortex volume and impaired cognitive function [52]. Older adults face an increased risk of mental disorders, such as depression and anxiety [53,54]. Aging has been associated with altered behavioral and molecular responses to chronic stress. For instance, middle-aged subordinate mice exposed to chronic stress exhibit heightened anxiety-like behaviors and amplified inflammatory responses, including elevated levels of IL6 and TNFα, compared with younger counterparts. These findings indicate that aging enhances vulnerability to chronic stress [55].

The CMS paradigm highlights mitochondrial dysfunction as a key factor in stress-related pathology. In mice subjected to CMS, mitochondrial respiration rates were inhibited, and mitochondrial membrane potential was disrupted in the hippocampus, cortex, and hypothalamus [56]. Furthermore, ultrastructural mitochondrial damage, including swelling and vacuolation, was evident in these brain regions [56]. CMS also led to a reduction in mitochondrial membrane potential (ΔΨm) and ATP levels in the rat prefrontal cortex [35].

### 5.2. Animal Models of Schizophrenia

Several animal models of schizophrenia have been developed, such as the maternal immune activation model, amphetamine models, phencyclidine (PCP) models, and DISC1 knock-out [57]; however, only one model, the polyinosinic-polycytidylic acid [poly(I:C)] model, has so far been used for mitochondrial transplantation [58,59], the details of which are described below.

#### Poly(I:C) Schizophrenia Model

Prenatal poly(I:C) immune challenge is a widely used developmental model to study schizophrenia-like pathology [60]. In this mouse model, pregnant dams are exposed to poly(I:C), a synthetic analog of viral double-stranded RNA, to mimic the immune response to viral infection. Poly(I:C)-induced maternal immune activation has been extensively used to model neurodevelopmental disorders such as schizophrenia. This model replicates key features of the disorder, with behavioral and cognitive abnormalities and heightened sensitivity to NMDA-receptor antagonists predominantly manifesting post-pubertally, which aligns with clinical observations in humans [61,62,63,64]. The pro-inflammatory cytokine surge during gestation disrupts fetal brain development, leading to behavioral and cognitive abnormalities later in life. The maternal immune activation model highlights how prenatal immune challenges contribute to neurodevelopmental alterations relevant to human disease. Poly(I:C) activates toll-like receptor 3 (TLR3), triggering the release of pro-inflammatory cytokines, including IL1β, IL6, and TNFα, as well as type I interferons (IFNα and IFNβ) [65,66,67,68]. This immune activation simulates the acute phase response to viral infection, enabling the effects of prenatal inflammation on brain development and behavior in offspring to be studied [67].

Furthermore, the poly(I:C) model can reveal functional and behavioral changes, as well as mitochondrial dysfunction, including reduced ΔΨm in frontal cortex neurons and altered transcript levels of respiratory complex subunits [58,69].

## 6. Mitochondrial Transplantation in Animal Models of Psychiatric Disorders

### 6.1. Mitochondrial Transplantation Methods and Timing

**Figure 1:**
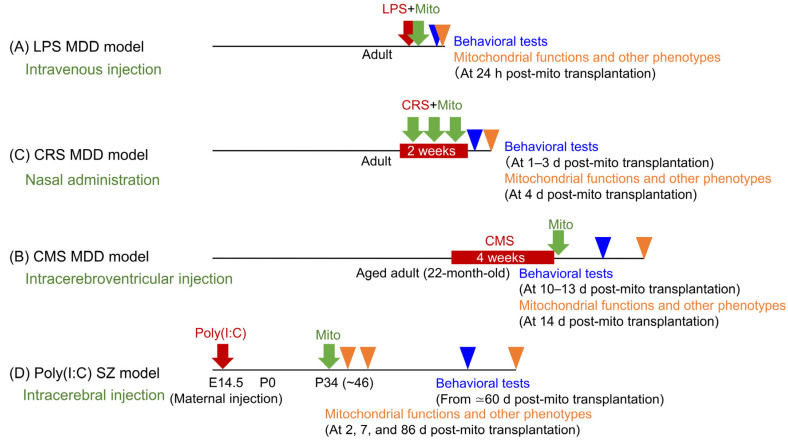
Experimental protocols for mitochondrial transplantation in animal models of psychiatric disorders. The red arrows or boxes indicate the time points of psychiatric disorder model treatments, the green arrows indicate the time points of mitochondrial transplantation, the blue arrowheads indicate the time points of behavioral tests, and the orange arrowheads indicate the time points of the analysis of mitochondrial functions and other phenotypes. LPS: Lipopolysaccharide; MDD: major depressive disorder; CRS: chronic restraint stress; CMS: chronic mild stress; Mito: mitochondria.

The methods of mitochondrial transplantation, timing of interventions, and intervals until behavioral assessments that have been used vary across models, as summarized below and in Table 1 and Figure 1.

#### 6.1.1. LPS MDD Model

Mitochondria were transplanted intravenously at the time of LPS injection. The effects of mitochondrial transplantation were evaluated at 16 and 24 h post-intervention. However, mitochondrial integration into the brain was not investigated [34].

#### 6.1.2. CRS MDD Model

Mitochondria were administered intranasally during the two-week CRS exposure period, with administration occurring three times every other day [33]. The effects of mitochondrial transplantation on behavioral phenotypes were assessed 1–3 days after the CRS treatment. Additionally, the effects of mitochondrial transplantation on biomarkers of mitochondrial function and neuroinflammation were evaluated after the behavioral tests. Mitochondrial integration into the brain was not examined [33].

#### 6.1.3. Aged CMS MDD Model

Mitochondria were delivered via intracerebroventricular injection immediately following four weeks of CMS exposure. Behavioral phenotypes were evaluated 10 days post-transplantation [35]. Histological and biochemical analyses were conducted 14 days after the transplantation [35].

#### 6.1.4. Poly(I:C) Schizophrenia Model

In this model, mitochondria were transplanted into the medial prefrontal cortex (mPFC) via intracerebral injection on postnatal day 34 or during the postnatal day 34–46 developmental period. This period is considered asymptomatic but responsive to antipsychotic drugs in poly(I:C)-exposed offspring [58,59]. Behavioral abnormalities were evaluated in adulthood, starting from postnatal day 90 or 95 [58,59]. Histological and biochemical measurements were performed two and seven days post-transplantation and again in adulthood, one week after the completion of behavioral testing [58,59].

### 6.2. Behavioral Improvements After Mitochondrial Manipulation

#### 6.2.1. MDD Models

Mitochondrial transplantation has shown promise in alleviating depressive-like behaviors across multiple MDD animal models. In the LPS-induced MDD model in rats, intravenous mitochondrial transplantation improved depressive-like behaviors, including reducing immobility time and alleviating stress-induced anhedonia, as measured by the forced swim test, tail suspension test, and sucrose preference test at 24 h post-intervention [34]. In the CRS MDD model in mice, intranasal mitochondrial transplantation demonstrated anxiolytic and antidepressant effects. Anxiety-like behaviors were reduced, as assessed by the elevated plus maze and open field test, while depressive-like behaviors were alleviated in the tail suspension test [33]. Similarly, in the aged CMS MDD model in rats, intracerebroventricular mitochondrial transplantation improved both anxiety- and depression-like behaviors. These effects were evaluated using the elevated plus maze, open field test, forced swim test, and sucrose preference test, indicating broad therapeutic potential [35].

#### 6.2.2. Schizophrenia Models

In the poly(I:C)-induced schizophrenia model, mitochondrial transplantation into the medial prefrontal cortex via intracerebral injection improved attentional deficits, as assessed by latent inhibition, and reduced hyperactivity, measured through spontaneous locomotor activity in a novel environment; both of these behaviors are linked to prefrontal cortex function. However, mitochondrial transplantation did not ameliorate impairments in social recognition or amphetamine-induced hyperactivity, which are primarily associated with orbitofrontal and striatal functions [58,59].

### 6.3. Possible Mechanisms of Mitochondrial Therapy

With respect to the underlying mechanisms of mitochondrial therapy, the LPS and CRS MDD models focus on the hippocampus, whereas the aged CMS MDD model and the poly(I:C) schizophrenia model target the prefrontal cortex (Table 1 and Table 2).

#### 6.3.1. LPS MDD Model

The intravenous injection of mitochondria reduced ROS levels and restored ATP production in the hippocampus to baseline, while normalizing the astrocyte and microglial activation induced by LPS [34]. Additionally, the mRNA levels of pro-inflammatory cytokines, including *IL1β*, *TNFα*, and *COX2*, in the hippocampus were restored to control levels [34]. Importantly, mitochondrial transplantation also reinstated neurogenesis in the dentate gyrus of the hippocampus to normal levels [34]. As adult-generated hippocampal neurons are essential for mood regulation and the efficacy of antidepressant treatments [70], these findings indicate that the therapeutic benefits of mitochondrial transplantation are mediated by the recovery of mitochondrial function, attenuation of neuroinflammation, and promotion of hippocampal neurogenesis.

#### 6.3.2. CRS MDD Model

Intranasal mitochondrial transplantation normalized corticosterone levels in the blood, reflecting a reduction in systemic stress hormone levels [33]. Consistent with findings in the LPS MDD model, mitochondrial transplantation reduced ROS levels and increased ATP production in the hippocampus [33]. Furthermore, it significantly decreased the protein levels of inflammatory markers, including IL1β, NLRP3, and the cleaved caspase 1/pro-caspase 1 ratio, in the hippocampus [33]. The activation of the NLRP3 inflammasome drives caspase 1 activation, leading to the production of pro-inflammatory cytokines IL1β and IL18, as well as pyroptosis, a rapid and inflammatory form of cell death [71,72,73]. Aberrant NLRP3 activation has been linked to mitochondrial dysfunction [74], and the NLRP3 inflammasome serves as a critical mediator between immune dysregulation and the development of depression [75]. Mitochondrial transplantation may, therefore, exert its therapeutic effects on CRS-induced depression by restoring mitochondrial function and suppressing the NLRP3/caspase 1/IL1β signaling pathway.

#### 6.3.3. Aged CMS MDD Model

Intracerebroventricular mitochondrial transplantation restored ΔΨm and ATP levels in the prefrontal cortex [35]. This intervention reduced the protein levels and activity of indoleamine 2,3-dioxygenase (IDO), the first and rate-limiting enzyme in tryptophan degradation, a critical step in the kynurenine pathway [35]. This was accompanied by a decrease in kynurenine levels, while tryptophan levels remained unchanged, indicating the modulation of the IDO/kynurenine pathway [35]. Furthermore, mitochondrial transplantation restored dendrite length, basal dendrite length, and spine density in the prefrontal cortex [35]. The activation of the IDO/kynurenine pathway plays a significant role in mood regulation and has been implicated in MDD and aging-related disorders [76,77,78]. A decrease in mitochondrial content or activity within dendrites is associated with the loss of synapses and dendritic spines [79]. These findings indicate that mitochondrial transplantation may alleviate depressive symptoms by restoring mitochondrial function and maintaining structural integrity in the prefrontal cortex.

#### 6.3.4. Poly(I:C) Schizophrenia Model

Mitochondrial transplantation into the medial prefrontal cortex via intracerebral injection restored dendrite branching and spine density in adulthood [59]. Within 2–7 days post-transplantation, acute mitochondrial manipulation improved mitochondrial function, including increased COX activity, elevated ΔΨm, and reduced ROS levels in the prefrontal cortex [59]. Neural activation was also enhanced, as indicated by the normalized c-FOS expression [59]. The transplantation further normalized the ratio of pro-inflammatory to anti-inflammatory cytokines, such as TNFα/IL10, indicating a rebalancing of the neuroimmune environment [59]. Additionally, a significant increase in microglial density (IBA1^+^ cells) was observed following mitochondrial manipulation. This was accompanied by attenuated cytokine levels, pointing to the role of microglia in synaptic reorganization, which may facilitate synaptic plasticity and the remodeling of neuronal networks [59,80]. These findings highlight the potential of mitochondrial transplantation to target mitochondrial dysfunction, neuroimmune imbalance, and structural deficits associated with schizophrenia-like pathology.

## 7. Transfer of Mitochondria into the Brain

Mafikandi et al. and Wang et al. conducted studies involving nasal and intravenous administration of mitochondria into the CRS MDD model and the LPS MDD model, respectively [33,34]. However, these studies did not verify the distribution of exogenous mitochondria within brain cells, leaving it unclear whether healthy mitochondria successfully migrated into the brain and functioned as a therapeutic intervention [33,34]. In contrast, previous studies have provided evidence supporting the distribution of exogenous mitochondria within various tissues. For example, Fu et al. demonstrated the in vivo distribution of exogenous mitochondria following intravenous injection in mice, using GFP-tagged mitochondria to trace their localization [81]. Within 2 h of administration, these mitochondria were detected in peripheral organs, including the liver, lungs, brain, muscles, and kidneys [81]. Additionally, Alexander et al. demonstrated that human mesenchymal stem cell-derived mitochondria administered nasally in mice rapidly reached the meninges within 30 min, where they were internalized by macrophages [82]. Their presence was validated using an anti-human mitochondria antibody and immunohistochemistry [82]. Furthermore, these mitochondria accessed the rostral migratory stream and various brain regions, including the hippocampus, where they colocalized with GFAP+ cells as early as 30 min post-administration and persisted for up to 18 h [82].

Javani et al. showed that, following intracerebroventricular injection, transplanted mitochondria were detected in the cells of the prefrontal cortex 14 days post-transplantation in the CMS MDD model [35]. The mitochondria were labeled with the mitochondrial-specific indicator MitoTracker, and fluorescence was detected in brain sections prepared using a freezing microtome and observed under a fluorescence microscope [35]. Similarly, mitochondrial transplantation into the medial prefrontal cortex via intracerebral injection revealed that exogenous mitochondria preferentially entered neurons, with fewer mitochondria in astrocytic processes and almost none in microglia in the Poly(I:C) SZ Model. For tracking, isolated active mitochondria were stained with MitoTracker, injected intracerebrally, and observed 3 h post-transplantation using confocal microscopy [59]. These differences in the types of cells internalizing exogenous mitochondria may be attributed to the transplantation method used, such as nasal administration versus intracerebral injection. Furthermore, differences in the cell types accessed might result in the activation of distinct mechanisms underlying the therapeutic effects of mitochondrial transplantation. Future studies are necessary to investigate these differences further and to clarify their implications.

## 8. Potential Risks of Mitochondrial Manipulation

A major concern regarding mitochondrial transplantation is the potential for side effects and complications [83]. Mitochondrial transplantation in animal models of psychiatric disorders has exclusively used allogeneic, not autologous mitochondria (Table 1) [33,34,35,58,59]. Healthy mitochondria transplanted into the medial prefrontal cortex of adolescent rats were beneficial in the poly(I:C) schizophrenia model; however, they were detrimental in healthy control rats [59]. Specifically, normal rats that received mitochondrial transplantation exhibited attentional deficits similar to those seen in the poly(I:C) schizophrenia model, including a behavioral and a dendritic abnormality [59]. Mitochondrial transplantation in normal rats induced a substantial increase in ROS and immune imbalance during the early post-transplantation phase, followed by an elevation in neuronal stress chemokine secretion [59]. Additionally, in adolescence and adulthood, similar patterns of protein-level changes in the prefrontal cortex were observed in both poly I:C-treated and normal rats that underwent transplantation [59]. This suggests that disruption to the late developmental stages of the prefrontal cortex contribute to long-term effects [59]. Ene et al. emphasize the need to consider potential detrimental effects arising from the dual nature of mitochondria, as well as their interaction with the physiological state, pharmacological treatments, immunological factors, and the inherent mitochondrial status of the recipient [59].

Recent research highlights the immunological challenges of allogeneic mitochondrial transplantation in animal models. For example, studies with deceased organ donors show that circulating mitochondria release damage-associated molecular patterns (mtDAMPs), triggering systemic inflammation and early graft dysfunction, mediated by cytokines like IL-6 and TNF-α [84]. Similar effects, including endothelial activation and increased adhesion molecule expression (e.g., ICAM-1, VCAM-1), were observed in murine heart transplantation, heightening graft rejection risk [85]. However, controlled experiments in BALB/cJ mice demonstrated minimal immune responses to syngeneic and allogeneic mitochondrial injections, indicating that outcomes depend heavily on mitochondrial preparation and host compatibility [86]. Autologous mitochondrial transplantation is thought to minimize the risk of inflammation and rejection because the mitochondria are derived from the patient’s own tissues. Animal studies have shown no significant increases in inflammatory markers following autologous transplantation, with no detection of anti-mitochondrial antibodies using the regional ischemia model [87]. Similarly, analyses of human peripheral blood mononuclear cells confirmed the absence of autoimmune or inflammatory responses associated with autologous transplantation [87]. A Phase 1 open-label, single-arm trial further confirmed the safety and feasibility of intra-arterial autologous mitochondrial transplantation during mechanical thrombectomy for acute ischemic stroke [88]. These findings provide insights that could inform mitochondrial transplantation strategies for psychiatric disorders, where immunological safety is a key concern.

Another critical concern associated with allogeneic mitochondrial transplantation is mitochondria–nucleus incompatibility, a phenomenon arising from genetic mismatches between donor mitochondrial DNA (mtDNA) and host nuclear DNA (nDNA). This mismatch disrupts vital cellular processes, including oxidative phosphorylation and ATP production, potentially leading to impaired cellular function and metabolic dysregulation [89,90]. In mouse models, introducing two genetically distinct mtDNAs has demonstrated genetic instability, leading to adverse physiological effects and behavioral changes. This highlights the risks of heteroplasmy and underscores the evolutionary advantage of uniparental mtDNA inheritance [91]. Despite these challenges, experimental models have demonstrated the potential for achieving compatibility under specific conditions. For instance, studies involving the transplantation of mouse or buffalo mitochondria into bovine oocytes have supported embryonic development to the blastocyst stage, suggesting that functional integration between donor mitochondria and host cells is achievable in controlled settings [92]. However, further research is necessary to clarify the long-term safety and functionality of such interventions, particularly regarding the stability of mixed mtDNA populations.

Allogeneic mitochondrial transplantation presents significant ethical challenges that must be addressed alongside its scientific and clinical hurdles. A key issue is mitochondria–nucleus incompatibility and the risk of transmitting pathogenic mutations through donor mtDNA, especially in germline cells, which could lead to intergenerational transmission and further complicate the ethical landscape [93]. Emerging techniques like advanced mitochondrial transfer, which avoids nuclear material transfer, may mitigate some ethical concerns, but challenges such as donor source selection and the ethical implications of cross-species mitochondrial use remain [93]. Comprehensive informed consent and long-term follow-up studies are critical to ensuring the safety and efficacy of these therapies while addressing evolving sociopolitical and ethical contexts [93].

## 9. Discussion

The specific mechanisms that are activated by mitochondrial transplantation in psychiatric disorder models remain unclear. However, the results of mitochondrial transplantation in these psychiatric disorder models commonly demonstrate restored mitochondrial function, such as increased ATP production and reduced ROS levels, along with decreased inflammation. These effects may represent key mechanisms underlying the therapeutic improvement of psychiatric disorders through mitochondrial transplantation [33,34,35,58,59].

Autologous rather than allogeneic mitochondrial transplantation is preferable because of its low risk of inflammation and rejection and ethical advantages. However, this approach is not suitable for patients with congenital mitochondrial disorders, because defective mitochondria may be present throughout the body. In such cases, allogeneic mitochondria from genetically similar donors are required. While some studies indicate that allogeneic mitochondria do not trigger alloreactivity or damage-associated molecular pattern responses, other studies report inflammatory activation, including endothelial cell activation and increased cytokine and chemokine expression [84,85,86]. Further research is essential to understand the immune mechanisms underlying allogeneic transplantation and to optimize its safety for broader clinical applications.

The timing of mitochondrial administration and the interval between transplantation and behavioral analysis in psychiatric disorder models vary and appear to be influenced by the delivery method [33,34,35,58,59] (Figure 1). For example, intravenous and nasal delivery methods are administered concurrently with stress exposure, potentially mitigating stress responses [33,34]. These minimally invasive approaches offer advantages for clinical application but may primarily serve a preventive role. A significant limitation of these methods in MDD models is that behavioral assessments are conducted only 24 h after mitochondrial transplantation, leaving their long-term effects unknown [33,34]. In contrast, mitochondrial transplantation in the schizophrenia models occurs approximately 40 days after maternal poly(I:C) injection—an asymptomatic period during which atypical antipsychotics can prevent brain and behavioral abnormalities in adult offspring [58,59,94]. In addition to the difference in transplantation regimen between aforementioned MDD models and schizophrenia models, the behavioral analyses of the schizophrenia models are conducted about 60 days post-transplantation (not 24 h), aligning the schizophrenia model approach more closely to clinical reality [58,59]. However, the delivery method for these studies involves intracerebral injection, which is highly invasive [58,59]. While intracerebral methods show therapeutic potential, their invasive nature presents challenges for clinical application, warranting careful evaluation in future research.

To optimize mitochondrial transplantation as a treatment for these disorders, it is essential to clarify the delivery routes, mechanisms of mitochondrial uptake, and to determine the cell types, such as glial cells, neurons, or other cell types, that preferentially take up mitochondria. The fate of mitochondria integrated into the brain is also of critical importance. A recent study using three-dimensional super-resolution microscopy and transmission electron microscopy revealed that isolated mitochondria are internalized by recipient cells and initially transported to endosomes and lysosomes [7]. Most of these internalized mitochondria eventually escape the endolysosomal system and integrate into the endogenous mitochondrial network [7]. It is proposed that multiple mechanisms may facilitate the internalization of exogenous mitochondria, highlighting the need for further research to clarify these pathways. Nonetheless, the long-term fate of the mitochondria that reach the brain remains uncertain, representing an important direction for future investigations.

## 10. Conclusions and Future Perspectives

The specific mechanisms involved in mitochondrial transplantation in various psychiatric disorders remain poorly understood, necessitating further research to develop therapeutic interventions aimed at modulating mitochondrial function. Currently, mitochondrial transplantation has been investigated exclusively in animal models of MDD and schizophrenia within the context of psychiatric disorder models. However, evidence of mitochondrial dysfunction in other psychiatric disorders, such as bipolar disorder [29,95,96], suggests that mitochondrial transplantation may have broader applications beyond MDD and schizophrenia. Future research will likely begin with experimental trials in animal models to assess its efficacy in these additional conditions. Animal models have been instrumental in uncovering the pathophysiology, progression, and potential treatments for psychiatric disorders. However, they cannot fully replicate the complexity of human conditions. To ensure their relevance, models should demonstrate face validity, construct validity, and predictive validity. Selecting models that meet these criteria enhances their translational value [97]. Therefore, it is essential to use a broad range of models to comprehensively explore the potential of mitochondrial transplantation. Additionally, the strategies for administration, including delivery methods and timing, must be further optimized to maximize therapeutic outcomes. The efficacy of mitochondrial transplantation has been demonstrated not only with autologous mitochondria but also with allogeneic and exogenous mitochondria. However, the latter approaches pose potential risks, such as immune rejection. Advances in techniques for modifying or regulating isolated mitochondria might help mitigate these risks and optimize transplantation protocols. Overall, research into the application of mitochondrial transplantation for psychiatric disorders remains in its early stages. Further preclinical and clinical studies are essential to fully understand its therapeutic potential, safety, and efficacy. The key challenges include optimizing mitochondrial isolation and delivery methods, understanding the fate of transplanted mitochondria, ensuring their long-term functionality, and addressing ethical concerns. Despite these challenges, mitochondrial transplantation holds significant promise as an innovative approach for targeting mitochondrial dysfunction and treating various psychiatric disorders, including psychotic conditions.

## Figures and Tables

**Table 1:** Summary of methods and techniques for in vivo studies of mitochondrial transplantation in animal models of psychiatric disorders.

Disorder	Treatment	Species	Source of Mitochondria	Route of Mitochondria Transplantation	Internalization in Brain Cells	Brain Area Analyzed	Ref.
MDD	LPS	Rat	Rat hippocampus (allograft)	Intravenous injection	NE	Hip	[34]
MDD	CRS	Mouse	Mouse brain (allograft)	Nasal administration	NE	Hip	[33]
Aged MDD	CMS	Rat	Young rat brain (allograft)	Intracerebroventricular injection	Yes	PFC	[35]
SZ	Poly(I:C)	Rat	Rat brain (allograft)	Intracerebral injection into the PFC	NE	PFC	[58]
SZ	Poly(I:C)	Rat	Rat brain (allograft)	Intracerebral injection into the mPFC	Yes (neurons)	PFC	[59]

MDD: major depressive disorder; SZ: schizophrenia; LPS: lipopolysaccharide; CRS: chronic restraint stress; Poly(I:C): polyinosinic-polycytidylic acid; CMS: chronic mild stress; Hip: hippocampus; PFC: prefrontal cortex; NE: not examined.

**Table 2:** Possible mechanisms of mitochondrial therapy.

Animal Model	Mechanisms in the Brain	Ref.
LPS MDD Model	- Restoration of ATP levels	[34]
	- Reduction in oxidative stress	
	- Reduction in neuroinflammation	
	- Enhancement of neurogenesis	
CRS MDD Model	- Restoration of ATP levels	[33]
	- Reduction in oxidative stress	
	- Reduction in neuroinflammation (IL-1β and NLRP3 pathways)	
	- Reduction in caspase-1 activation	
CMS MDD Model	- Restoration of mitochondrial membrane potential (ΔΨm) and ATP production	[35]
	- Reduction in IDO activity and kynurenine (Kyn) levels	
	- Enhancement of dendritic spine density and dendritic length	
Poly(I:C) SZ Model	- Promotion of ΔΨm	[58,59]
	- Reduction in oxidative stress	
	- Modulation of neuroinflammatory responses	

MDD: major depressive disorder; SZ: schizophrenia; LPS: Lipopolysaccharide; CRS: chronic restraint stress; CMS: chronic mild stress.

## Data Availability

Not applicable.
